# A Mechanistic *In Vivo*/*Ex Vivo* Pharmacokinetic‐Pharmacodynamic Model of Tenofovir for HIV Prevention

**DOI:** 10.1002/psp4.12583

**Published:** 2021-02-06

**Authors:** Priya Jayachandran, Maria Garcia-Cremades, Katarina Vučićević, Namandjé N. Bumpus, Peter Anton, Craig Hendrix, Radojka Savić

**Affiliations:** ^1^ Department of Bioengineering and Therapeutic Sciences University of California San Francisco San Francisco California USA; ^2^ Department of Pharmacokinetics and Clinical Pharmacy Faculty of Pharmacy University of Belgrade Belgrade Serbia; ^3^ Division of Clinical Pharmacology Department of Medicine Johns Hopkins University Baltimore Maryland USA; ^4^ University of California Los Angeles Los Angeles California USA

## Abstract

Defining tissue and plasma‐specific prophylactic drug concentrations is central to pre‐exposure prophylaxis product development for sexual transmission of HIV‐1. Pharmacokinetic (PK) data from study RMP‐02/MTN‐006 comparing single dose oral tenofovir disoproxil fumarate with single and multiple dose rectal tenofovir (TFV) gel administration in HIV‐1 seronegative adults was used to construct a multicompartment plasma‐rectal tissue population PK model for TFV and tenofovir‐diphosphate (TFVdp) in plasma and rectal tissue. PK data were collected in five matrices: TFV (plasma, rectal tissue homogenate), TFVdp (peripheral blood mononuclear cells, rectal mononuclear cells (MMCs), rectal tissue homogenate). A viral growth compartment and a delayed effect compartment for p24 antigen expression measured from an *ex vivo* explant assay described HIV‐1 infection and replication. Using a linear PK/pharmacodynamic model, MMC TFVdp levels over 9,000 fmol/million cells in the explant assay provided apparent viral replication suppression down to 1%. Parameters were estimated using NONMEM version 7.4.

**Study Highlights**

**WHAT IS THE CURRENT KNOWLEDGE ON THE TOPIC?**

*Ex vivo* explant assays provide a dynamic and quantitative measurement of the cumulative p24 antigen expression level following HIV‐1 infection over an assay time period. They have been used in a descriptive and nonmechanistic way to characterize HIV‐1 infection dynamics.

**WHAT QUESTION DID THIS STUDY ADDRESS?**

With consideration for viral dynamics and drug degradation over time, what is the intracellular tenofovir‐diphosphate (TFVdp) concentration in rectal tissue that is needed to suppress HIV‐1 infection as measured by an *ex vivo* explant challenge?

**WHAT DOES THIS STUDY ADD TO OUR KNOWLEDGE?**

The pharmacokinetic (PK)/pharmacodynamic model provides a novel mechanistic link between tissue TFVdp PK and virus kinetics by way of p24 antigen expression levels over time. A target intracellular drug concentration that provides apparent suppression of viral replication down to 1% for a rectal pre‐exposure prophylaxis (PrEP) microbicide is estimated.

**HOW MIGHT THIS CHANGE DRUG DISCOVERY, DEVELOPMENT, AND/OR THERAPEUTICS?**

It will inform the rational design and development of novel topical PrEP formulations by providing a quantitative framework to relate site‐specific drug concentrations to HIV‐1 infection suppression.

Recent therapeutic advances have revolutionized how HIV‐1 is treated and prevented. Adherence to antiretroviral treatment regimens typically leads to normal life expectancy, and adherence to pre‐exposure prophylaxis (PrEP) regimens results in low rates of new infection.^1^, ^2^, ^3^, ^4^, ^5^ Tenofovir (TFV) prodrugs are recommended as part of combination antiretroviral treatment regimens and are proven effective for prophylaxis with emtricitabine.^6^, ^7^, ^8^, ^9^, ^10^, ^11^, ^12^ TFV disoproxil fumarate (TDF), a prodrug, is hydrolyzed to TFV and subsequently phosphorylated intracellularly to TFV diphosphate (TFVdp).^13^ TFVdp is the active form of this nucleoside reverse transcriptase inhibitor that leads to virally encoded DNA chain termination.^13^

PrEP is recommended for individuals at high risk for becoming infected with HIV‐1.^14^ In the randomized clinical trials demonstrating oral PrEP efficacy (iPrEx, Partners Prep, TDF2, Bangkok Tenofovir Study), there is an increased relative risk reduction when outcomes are adjusted for adherence using objective pharmacologic measures (e.g., plasma TFV concentrations).^7^, ^9^, ^10^, ^11^ Given the challenges of adhering to daily oral PrEP indicated by poor adherence in some studies, alternative formulations are in development, including long‐term strategies to minimize the need for strong adherence, and “on‐demand” approaches that only require adherence during periods of sexual risk.^15^ These alternative systemic and topical formulations (e.g., intravaginal ring and vaginal gel) may help overcome barriers associated with oral PrEP and provide a greater breadth of formulations to choose from.

For PrEP candidates, the target drug exposure necessary to prevent HIV‐1 infection in plasma, peripheral blood mononuclear cells (PBMCs), and mucosal mononuclear cells (MMCs) remains unclear.^16^ Further, target concentrations may differ by exposure route (e.g., oral or rectal).^16^ Through developing pharmacokinetic/pharmacodynamic (PK/PD) models to better understand the relationship between drug exposure and infection suppression after systemic and topical administration, we hope to inform how specific administration routes may be tailored to achieve protective concentrations at key sites of action.

To explore this PK/PD relationship for topical PrEP, several studies have investigated TFV formulated as a microbicide gel applied vaginally or rectally.^8^, ^17^, ^18^ Study RMP‐02/MTN‐006 evaluated the PK of TFV 1% vaginal gel applied rectally compared with oral 300 mg TDF in HIV‐1 seronegative adults.^18^ More significant accumulation of TFVdp than TFV occurred in rectal tissue likely due to the longer (60 hours) intracellular half‐life of TFVdp compared with the shorter (31 hours) tissue TFV half‐life.^19^, ^20^ Excised rectal tissue was challenged *ex vivo* with HIV‐1, and the extent to which HIV‐1 growth was suppressed in these infected tissue explants, denoted by a decline in p24 antigen expression, was used as a biomarker for microbicide efficacy.^21^ The p24 is an HIV‐1 capsid protein that is detectable in the serum in the early stages of infection before the HIV‐1 antibody is detected.^22^ TFVdp was more predictive of *ex vivo* HIV‐1 growth inhibition than TFV.^21^ A logistic regression statistical model revealed a decline in cumulative p24 antigen expression that was nonsignificant following single dosing of either the oral or rectal formulation, but significant following multiple rectal dosing due to higher concentrations achieved intracellularly with multiple dosing.^21^ Effective concentration (EC)_50,90,95_ levels were predicted as an efficacy end point for *ex vivo* infection suppression, but disease progression over time (reflected by the p24 antigen expression‐time profile) with consideration for p24 variability was not characterized.

We developed a mechanistic *in vivo/ex vivo* population PK/PD model to characterize the baseline viral growth, viral growth‐response of TFV in *ex vivo* explant tissue, and p24 variability. The model was used to propose *in vivo* tissue‐specific protective levels of TFVdp that may prevent HIV‐1 infection in the explant challenge. Our findings provide a quantitative and mechanistic framework for understanding the PK/PD relationship central to topical PrEP drug development, and may be used to inform future product development (e.g., rectal enema).

## METHODS

### Study population design

**Figure 1:**
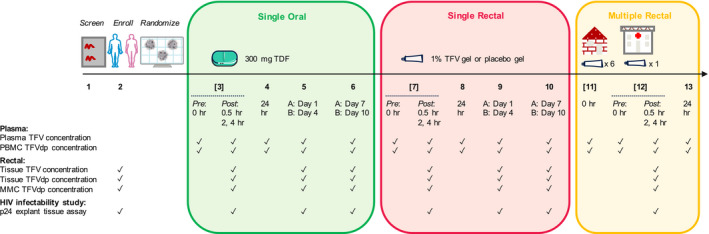
Schematic of RMP‐02/MTN‐006 clinical study. Collection times following oral tablet and rectal gel administration per study arm for (1) parent and metabolite drug concentration in plasma and rectal matrices and (2) p24 antigen level from HIV‐1 infectibility study. TDF, tenofovir disoproxil fumarate; TFV, tenofovir; TFVdp, tenofovir‐diphosphate.

The full study design of RMP‐02/MTN‐006 is published elsewhere (NCT00984971).^18^, ^19^, ^21^ The trial was approved by the institutional review board at each site (University of California at Los Angeles, Los Angeles, CA and Magee‐Women’s Research Institute, Pittsburgh, PA). In brief, it is a two‐site, phase I, partially blinded, placebo‐controlled safety, acceptability, and PK trial of TFV 1% vaginal gel (44 mg TFV) applied rectally compared with oral 300 mg TDF (136 mg TFV) in HIV‐1 seronegative adults.^18^, ^19^ Eighteen healthy volunteers received a single dose of oral TDF (arm 1, single oral). A month later, subjects were randomized (2:1) to receive either a single rectal dose of TFV 1% or hydroxyethylcellulose (placebo) gel (arm 2, single rectal). A month after arm 2 concluded, subjects received 7 consecutive doses (home, 6; clinic, 1) of either gel (arm 3, multiple rectal).^18^ **Figure** **1** illustrates the study design.

### Pharmacokinetic sampling

Plasma samples were collected from all individuals throughout each arm of the study from 30 minutes through 14 days after each single exposure and from 30 minutes through 24 hours following 7‐day rectal exposure.^19^ TFV concentrations in two compartments (plasma and rectal tissue homogenate) and TFVdp in three compartments (PBMCs, rectal tissue homogenate, and MMCs) were quantified.^21^ Rectal tissue biopsies were collected postdose in the single exposure arms on days 1 and 7 or 4 and 10 depending on the randomization group and multiple rectal arm at 30 minutes. Compartment concentrations and explant tissue samples were collected simultaneously at visits 2 (baseline), 3, 5, 6, 7, 9, 10, and 12.^21^ **Figure** **1** illustrates the collection times for parent and metabolite compartment concentrations measured per matrix and the HIV‐1 infectibility study at each visit.

### General structural model development

**Figure 2:**
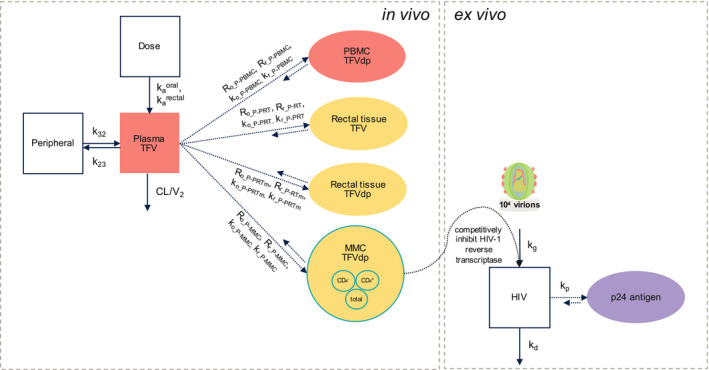
Schematic of mechanistic population pharmacokinetic/pharmacodynamic (PK/PD) model. (Left, *in vivo*) Multicompartment PK model with a plasma compartment and effect compartments for each cellular (PBMC and MMC) and tissue (rectal tissue homogenate) matrix. (Right, *ex vivo*) Mechanistic PK/PD model with a viral growth compartment and a delayed compartment for p24 antigen expression. TFV conversion to TFVdp occurs in MMCs resulting in inhibition of HIV‐1 growth and reduction of p24 antigen expression intracellularly following *ex vivo* infection with HIV‐1. CL, clearance; k_a_, drug absorption rate constant; k_d_, death rate; k_g_, growth rate; k_o_, uptake rate following oral administration; k_p_, p24 antigen expression rate; k_r_, uptake rate following rectal administration; m, metabolite; MMC, rectal mononuclear cell; P, plasma; PBMC, peripheral blood mononuclear cell; pink, plasma PK compartments; R_o_, ratio following oral administration; R_r_, ratio following rectal administration; TFV, tenofovir; TFVdp, tenofovir‐diphosphate; V_2_, central volume; V_3_, peripheral volume; yellow, rectal PK compartments.

A population approach was used to build the mechanistic PK/PD model (**Figure** **2**) linking a multicompartment PK model for plasma with biophase effect compartments for each matrix (*in vivo*) to a viral growth compartment with a delayed compartment for p24 antigen expression (*ex vivo*). Modeling was performed with NONMEM (version 7.4; ICON PLC, Dublin, Ireland). Data were fitted using the first‐order conditional estimation method with interaction except when handling data below the lower limit of quantification (LLOQ) of the assay (below limit of quantification (BLQ)). Diagnostic plots (goodness‐of‐fit and individual prediction) and objective function value (OFV) changes were used to evaluate each model iteration. Visual predictive checks (VPCs) using 500 simulated datasets were performed to assess model appropriateness and suggest improvements. Perl‐speaks‐NONMEM (PsN, version 4.7, 4.8), Pirana (version 2.9.7), R (version 3.5.1), and Xpose4 (version 4.6.1) were used for data management, model development, and data visualization.^23^

### Population pharmacokinetic model development

#### Plasma matrix

Plasma concentrations following oral and rectal administration were modeled separately, then jointly. A one‐compartment and two‐compartment model with oral absorption were fitted to the data with and without a lag parameter. A rectal absorption rate constant and bioavailability (relative to the oral route) were added. Initial estimates were based on values obtained from a noncompartmental analysis.^19^ Interindividual variability (IIV, η) was added to each PK parameter and assessed for model stability and convergence; IIV for k_a_^oral^ and V_2_/F was retained. A proportional (oral) and combined additive and proportional (rectal) error model described the residual variability.

#### Tissue and cellular matrices

To the plasma PK model, tissue and cellular matrices were added using a biophase effect compartment approach^24^:

(1) $$\frac{dC_{\mathrm{MMC}}}{\mathrm{dt}}=k_{P‐MMC}\left(R_{P‐MMC}C_{P}‐C_{\mathrm{MMC}}\right)$$

(2) $$\frac{dC_{\mathrm{PBMC}}}{\mathrm{dt}}=k_{P‐PBMC}\left(R_{P‐PBMC}C_{P}‐C_{\mathrm{PBMC}}\right)$$

(3) $$\frac{dC_{\mathrm{RT}}}{\mathrm{dt}}=k_{P‐RT}\left(R_{P‐RT}C_{P}‐C_{\mathrm{RT}}\right)$$

(4) $$\frac{dC_{\mathrm{RTm}}}{\mathrm{dt}}=k_{P‐RTm}\left(R_{P‐RTm}C_{P}‐C_{\mathrm{RTm}}\right)$$

where k_P-tissue/cellular matrix_ is the rate of drug entry into the tissue or cellular matrix from the plasma and R_P‐tissue/cellular matrix_ is a concentration ratio between the tissue or cellular matrix and plasma. The final plasma PK parameter estimates were fixed, whereas the rate and ratio of TFV distribution into rectal tissue and TFVdp distribution into rectal tissue, MMCs, and PBMCs were estimated. Initial estimates were based on the raw data. Both rate and ratio terms were stratified by administration route (oral: k_o_plasma‐tissue/cellular matrix_, R_o_plasma‐tissue/cellular matrix_; rectal: k_r_plasma‐tissue/cellular matrix_, R_r_plasma‐tissue/cellular matrix_), and reported in their original units. For PBMCs, a rectal rate and ratio term was not estimated because the concentrations in the single and multiple rectal arms were BLQ. Due to data sparsity in the tissue matrices, a sensitivity analysis was performed on the rate terms to determine if the uptake was either slow or fast. On the basis of model stability, convergence, OFV, and VPCs, the rates were fixed to either a “low” or “high” value indicating either a slow or fast uptake, respectively. IIV was added to the ratio term for each tissue and cellular matrix, and a proportional error model was used for each tissue or cellular matrix to assess the residual variability. For plasma, the LLOQ was 0.3 ng/mL; for the tissue and cellular matrices, each sample had its own unique LLOQ value based on cell count or biopsy mass and drug assay sensitivity per sample. The likelihood that observations deemed BLQ were less than the LLOQ (M3) was modeled with the Laplacian estimation method (not reported).^25^

### Pharmacodynamic sampling

Explant tissue samples were collected at baseline (visit 2) and postdose in the single exposure arms on days 1 and 7 or 4 and 10 depending on the randomization group (visits 3, 5, 6, 7, 9, and 10) and multiple rectal arm at 30 minutes postdose (visit 12).^21^ These samples were infected *ex vivo* with HIV‐1 within 1–2 hours of harvesting.^21^ Supernatants were collected on days 0 or 1, 4, 7, 11, and 14 to quantify p24 antigen levels.^21^ Measurements across 4 biopsies were averaged and added to the p24 antigen concentration from the previous time point to provide a cumulative level at each post‐challenge timepoint.

### Population PK/PD model development

#### PK/PD baseline model

Initially, baseline (absence of drug) p24 antigen expression characterizing viral replication and growth was described using linear, exponential, shifted logistic, 3‐parameter Gompertz, 4‐parameter Weibull, and mechanistic structural models. The mechanistic model was chosen due to its resemblance to the physiological system and was developed from a viral dynamics model.^26^ It consists of an exponential HIV‐1 viral growth model with a death rate and a delayed effect compartment describing the rate of p24 antigen expression:

(5) $$\frac{\mathrm{dV}}{\mathrm{dt}}=k_{g}V\left(1‐E\right)‐k_{d}V$$

(6) $$\frac{\mathrm{dP}}{\mathrm{dt}}=k_{p}\left(RV‐P\right)$$

where V is HIV‐1 virus (virions/mL), P is p24 antigen level (pg/mL), k_g_ is viral growth rate (/hour), k_d_ is viral death rate (/hour), k_p_ is p24 antigen expression rate (/hour), E is drug treatment effect, and R is ratio of virus to p24 antigen level. Assumptions included a negligible immune effect and constant k_p_.^26^ Both k_d_ and R were estimated while k_g_ and k_p_ were fixed to literature values of 0.045/hour and 0.0059/hour, respectively.^27^, ^28^ Residual variability was described by a combined additive and proportional error model. IIV was added to each rate term; IIV on k_p_ was fixed for modeling the treatment effect. Initial conditions were 10^4^ virions for V and zero for P.

#### PK/PD model with drug degradation effect

The PK/PD relationship for the MMC matrix was modeled using three relationships: linear, maximum effect (E_max_), and sigmoid E_max_. The simplest relationship (linear) that characterized the data well was selected based on OFV, diagnostic plots, and VPCs. The drug treatment effect was modeled as E = SL * CP where SL is slope (pg/mL)/(fmol/million cells) and CP is MMC TFVdp concentration (fmol/million cells). Two modes were explored: inhibition of growth (1‐E) and acceleration of death (1+E). Similar results were obtained for both models; the former was selected based on TFV mechanism of action. Explant tissue collected 30‐minutes postdose administration was linked to the MMC TFVdp concentrations measured at 30 minutes postdose for the exposure‐response relationship. The drug degradation time course during the explant assay was not studied; therefore, a constant rate of 0.0018/hour was assumed based on TFV drug degradation kinetic tests and modeled with an exponential decay.^29^ All other parameter estimates were fixed to the final estimates from the baseline model and SL was estimated. The explant assay LLOQ was 10 pg/mL; all BLQ observations were modeled using the M5 method.^25^

### Simulations to determine target tenofovir concentrations to suppress HIV‐1 infection

Using the PK/PD model, simulations were performed using the PKPDsim R package (version 1.0.1) to determine the target TFVdp concentration in MMCs necessary to achieve apparent viral replication suppression down to 1% in the explant assay. Viral replication trajectories were simulated for 2 weeks over a concentration range of 0 to 11,000 fmol/million cells; the lower end emulates the expression profile in the raw data, whereas the higher end describes the expected viral replication trajectory that may suppress HIV‐1 infection. Apparent viral replication suppression down to 1% was calculated using the linear relationship for drug treatment effect, E = k_g_(1‐SL*CP). When no drug is present, the effect captures the uninhibited growth of the virus. Using 1% of the maximum effect, the MMC TFVdp concentration necessary for apparent viral replication suppression down to 1% was calculated.

## RESULTS

### Study population data

Eighteen subjects (14 men and 4 women) aged 22 to 66 years old provided drug concentration levels in plasma (TFV), PBMC (TFVdp), rectal tissue homogenate (TFV and TFVdp), and MMC (TFVdp) matrices (**Figure** **1**). After dose administration, subjects provided rectal tissue for *ex vivo* HIV‐1 infection from which p24 antigen levels were measured across the explant assay time course. The total number of PK samples for each matrix and cumulative p24 antigen observations for each study arm are shown in **Table** **S1**; measured concentrations that are BLQ are denoted as a percentage per matrix.

### Population pharmacokinetic model

**Figure 3:**
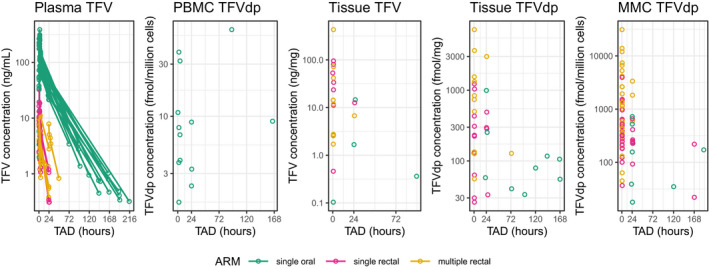
Pharmacokinetic profiles of raw data. Parent and metabolite drug concentration‐time profiles stratified by matrices and study arm. Concentrations deemed below the limit of quantification are not included. fmol, femtomole; mg, milligram; mL, milliliter; MMC, rectal mononuclear cell; ng, nanogram; PBMC, peripheral blood mononuclear cell; TAD, time after dose; TFV, tenofovir; TFVdp, tenofovir‐diphosphate.

A multicompartment PK model consisting of 7 compartments (**Figure** **2**) described the TFV and TFVdp PK time course in plasma and tissue/cellular matrices (**Figure** **3**). The plasma PK was described by a two‐compartment model with oral absorption. Biophase effect compartments for each tissue and cellular matrix for parent and metabolite drug concentrations were added. **Table** **1** provides the PK parameter estimates with relative standard errors per matrix. The model described the data well; owing to data sparsity (**Table** **S1**) in the tissue and PBMC matrices, VPCs for only the key matrices (plasma and MMC) stratified by study arm are shown in **Figure** **S1**. Applying the M3 method to BLQ observations resulted in parameter estimates with large relative standard errors (not shown) likely due to the large percentage of BLQs in the matrices (**Table** **S1**).

### Population PK/PD model

**Figure 4:**
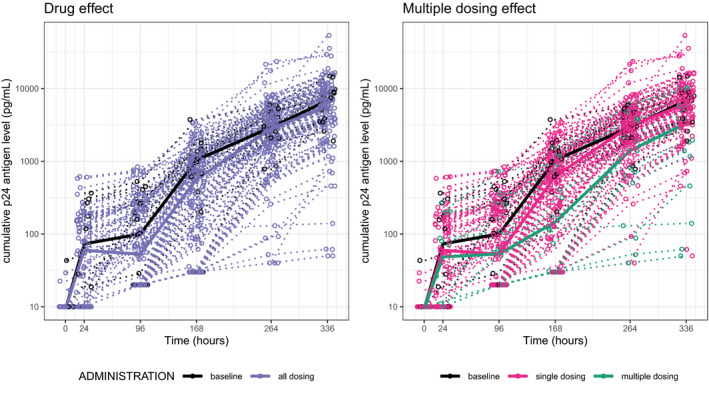
Pharmacodynamic profiles of raw data. Cumulative p24 antigen expression profiles stratified by [left] drug administration compared with baseline (no treatment) and [right] single (single oral, single rectal)/multiple (multiple rectal) drug administrations compared to baseline. Concentrations deemed below the limit of quantification (10 pg/mL) are included. mL, milliliter; pg, picogram.

The cumulative p24 antigen expression time course across the explant assay is shown in **Figure** **4**, and further stratified by PK percentile for each MMC type in **Figure** **S2**. For higher TFVdp concentrations, greater expression suppression is seen, which is captured by the slope of the colored lines (compared with baseline). The cumulative p24 antigen levels as a function of drug concentration are similar regardless of MMC type, which is expected given the high correlation of drug concentrations between each MMC type (**Figure** **S3**).

The p24 antigen expression was modeled for CD_4_^+^, CD_4_^−^, and total cells using a PK/PD model. The parameter estimates are provided for the baseline model (**Table** **S2**) and final model (**Table** **2**). The final model described the data well and no significant misspecifications were observed from the goodness‐of‐fit plots or the VPCs stratified by study arm (**Figure** **S4**). Accounting for the decline in drug concentration across the assay time course, a significant decrease in OFV relative to the “PK/PD effect” was observed for the “drug degradation effect” model, and the PK/PD effect was significant for all cell types (**Table** **S3**). Cell separation did not affect the assessment of drug efficacy, as evidenced by the similar value for the slope term in the drug degradation effect model for each cell type.

**Table 2:** PK/PD drug degradation model parameter estimates

Parameter	Population estimate (RSE, %)			IIV, %CV (RSE, %)		
	CD_4_^−^	CD_4_^+^	TOTAL	CD_4_^−^	CD_4_^+^	TOTAL
k_growth_ [/hour]	0.0320^a^	0.0320^a^	0.0320^a^			
k_death_ [/hour]	0.0193^b^	0.0193^b^	0.0193^b^			
k_p24_ [/hour]	0.00400^a^	0.00400^a^	0.00400^a^	72.5^b^	72.5^b^	72.5^b^
Ratio [pg/virions]	0.0404^b^	0.0404^b^	0.0404^b^			
k_degradation_ [/hour]	0.0018^a^	0.0018^a^	0.0018^a^	‐	‐	‐
Slope [(pg/mL)/(fmol/million cells)]	0.00042 (9)	0.00008 (44)	0.00011 (11)		171.2^c^	
Proportional error [%CV]	86.9 (5)	85.7 (6)	87.6 (6)	‐	‐	‐
Additive error [pg/mL]	3.53 (10)	3.60 (10)	3.61 (11)	‐	‐	‐

Parameter estimates for the pharmacokinetic‐pharmacodynamic model with drug degradation effect (final model) stratified by MMC type (CD_4_^‐^, CD_4_^+^, total).

%CV, percent coefficient of variation; IIV, interindividual variability; MMC, rectal mononuclear cell; PD, pharmacodynamic; PK, pharmacokinetic; RSE, relative standard error.

^a^ Estimate fixed to literature value.

^b^ Estimate fixed from baseline model.

^c^ Estimate fixed from previous iteration of model (PK/PD model).

### Target tenofovir concentrations to suppress HIV‐1 infection

**Figure 5:**
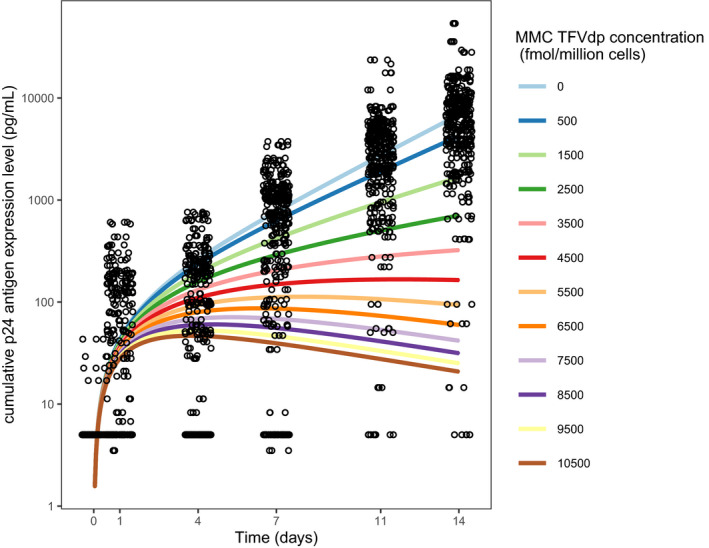
Simulated target MMC TFVdp concentration for apparent HIV‐1 replication suppression. Simulation of cumulative p24 antigen expression profiles over the assay time course (14 days) using the total MMC cell type pharmacokinetic‐pharmacodynamic model with drug degradation effect (final model). Raw p24 antigen expression data are superimposed (black circles). fmol, femtomole; mL, milliliter; MMC, rectal mononuclear cell; pg, picogram; TFVdp, tenofovir‐diphosphate.

Given the high correlation of TFVdp concentration across MMC types, the final model for the total cell type was used to perform simulations to determine a target MMC TFVdp concentration to obtain an apparent suppression of viral replication down to 1% in the explant assay (**Figure** **5**). The simulated viral growth trajectories at low concentrations (e.g., 500 fmol/million cells) mimic the p24 antigen expression profiles of the raw data. At higher concentrations (above 5,500 fmol/million cells), the slope plateaus and then trends downward. This change in virus trajectory suggests a failure to sustain replication, eventually leading to suppression. A target concentration of 9,000 fmol/million cells is required for apparent suppression of viral replication down to 1% for the explant challenge.

## DISCUSSION

The pivotal oral PrEP efficacy trials identified the negative impact poor adherence has on HIV‐1 prevention. Topical PrEP formulations used episodically represent one strategy to overcome this hurdle. A development challenge is ensuring there is a high enough active drug concentration (TFVdp) for a long enough time period within the target cells in the rectal mucosa. PK/PD modeling of data from completed oral PrEP efficacy trials can guide development of these equally efficacious topical formulations that achieve low systemic drug concentrations. Using data from study RMP‐02/MTN‐006, we built a mechanistic *in vivo/ex vivo* PK/PD model using nonlinear mixed effects modeling to quantify a dose‐concentration‐response relationship and propose a target TFVdp concentration in colorectal tissue cells from an explant challenge. The multicompartment PK model characterizes TFV and TFVdp distribution in plasma and at local sites of infection and drug action with estimates of typical values for key PK parameters (CL, V, k_a_, and F) per administration route and with consideration for hierarchies of variability (interindividual and residual). The *in vivo* MMC TFVdp concentration linked to *ex vivo* p24 antigen levels captures the basic viral dynamics of HIV‐1 growth and its suppression by TFV over time. The target TFVdp concentration established using an explant challenge model holds high value in prevention trials where viral challenges cannot be performed.

Several assumptions were used to create the PK/PD model. A negligible immune cell effect (given the absence of ingress/egress of blood/immune cells) contributing to virus death and a constant rate of p24 antigen production and viral growth were assumed to reduce the complexity of HIV‐1 viral dynamics. The model presents the worst‐case scenario in which any reduction in viral growth is solely due to the drug effect, not a combination of a drug and immune effect. Furthermore, the trajectory of p24 antigen expression closely matches that of HIV‐1 viral load and the levels are detectable 10–15 days after infection.^28^, ^30^ Therefore, a delayed effect compartment was assumed to capture p24 antigen expression after tissue infection (Eq. 6).

Despite lower plasma TFV levels, topical rectal administration yielded faster absorption into plasma and higher TFVdp levels in rectal tissue and MMCs compared with systemic oral administration. Rapidly achieving high intracellular TFVdp concentrations in target rectal mucosal cells following rectal administration results in faster virus suppression with less total drug exposure than via the oral route. This suggests that topical rectal PrEP may overcome the need to dose oral drugs in advance of potential rectal HIV‐1 exposure while also achieving higher target tissue concentrations when used in an “on‐demand” regimen. Therefore, collecting rectal tissue in addition to plasma in phase I studies is critical, and is now done routinely. By correlating a tissue with a plasma prophylactic concentration using the effect compartment model, the estimate of a preventive plasma concentration, which likely differs for oral compared with topical dosing, might be determined.

The multicompartment PK model is data‐informed; it does not describe intracellular TFV metabolism or the precise direction of drug movement after oral or rectal administration because data to support these relationships are unavailable. Drug movement is guided by central (plasma) and local (capillary) blood systems, and, over time, the concentration equilibrates between the plasma and rectal tissues. The effect compartment model provides an estimate of a relative ratio that describes this drug movement between the plasma and tissue/cell while making minimal assumptions. This simple model structure is useful to inform the PK given the data sparsity and is preferable to prior noncompartmental analyses that cannot describe the aforementioned relationships adequately.

Richardson‐Harman *et al*. estimated an explant TFVdp EC_90_ of 1,318, 13,183, and 8,313 fmol/million cells for CD_4_^−^, CD_4_^+^, and total MMC cell types, respectively.^21^ The EC_90_ for total MMCs is close to our proposed target MMC TFVdp concentration of 9,000 fmol/million cells estimated from simulations using the PK/PD model. In the DREAM‐01 study evaluating the PK of three formulations of TFV enema in men, an explant EC_50_ of 2,000 and EC_90_ of 20,000 fmol/million cells has been reported from preliminary analyses.^31^ By contrast, the PK/PD relationship between PBMC TFVdp concentration and HIV‐1 seroconversion in the iPrEx study provided a clinical IC_90_ of 40 fmol/M PBMC (95% confidence interval: 8–70) and IC_99_ of 53 fmol/M PBMC (95% confidence interval: 15–150).^32^, ^33^ In the HPTN 066 prevention study, which used directly observed dosing, these PBMC concentrations were consistent with daily TDF dosing and a median colon tissue cell TFVdp concentration of 160 fmol/million cells as a clinical 90% inhibitory concentration (IC_90_) correlate for rectal mucosa. Taken together, these estimates indicate a > 50‐fold difference between clinical and explant IC_90_ estimates.

Some of the apparent difference between the clinical and explant IC_90_ may be explained by limitations of the explant challenge study. The assay uses a high inoculum for infection and crude sample preparation techniques that are not reflective of clinical infection through receptive anal intercourse. The traumatic removal of rectal tissue using biopsy forceps is greater and deeper than coital trauma disruption and exposes an unprotected tissue surface that lacks the protective mucus and epithelial layers, likely enhancing the infectibility of the biopsy and resulting in the need for higher drug concentrations to fully prevent HIV‐1 infection. Additionally, isolating the simulated HIV‐1 challenge to a small colon biopsy detached from the rest of the body does not address those virions escaping mucosa to lymph nodes and the systemic circulation.

The PK/PD model has some limitations. Firstly, the study population was limited to 18 subjects. A large percentage of the observations were BLQ for tissue and cellular matrices, particularly the PBMC matrix, which may be due to low TFVdp exposure in the plasma after rectal dosing. The PK collection times in the trial were identical for oral and rectal administration; however, the rectal formulation had a lower dose (44 mg) and bioavailability (~ 10%) compared with the oral formulation. Therefore, the trial design was predisposed for a higher percentage of BLQs following rectal administration. Additionally, the cumulative p24 antigen levels were linked to MMC TFVdp concentrations at a single time point (30 minutes postdose corresponding to the time of explant infectivity). Lastly, during the 14‐day assay, the tissue cells’ ability to sustain viral replication was not measured nor was the time course of TFVdp concentration quantified. Future studies should measure the drug degradation effect on viral growth dynamics and replication sustainability to derive unbiased model parameter estimates and maximize the utility of the explant assay.

In conclusion, we developed a quantitative framework for understanding a central PK/PD relationship in topical PrEP product development, and we identified a target *ex vivo* tissue‐specific prophylactic TFVdp concentration. The approach may be applied to future PrEP product studies that correlate *ex vivo* tissue infectibility with plasma and tissue drug concentration after *in vivo* application of a systemic or topical product for any cellular/tissue compartment for which PK data are collected irrespective of the exposure route. Future directions include studying the relationship between *ex vivo* and *in vivo* target intracellular prophylactic concentrations using PrEP clinical trial data to further re‐align data from *ex vivo* explant assays with effective *in vivo* target TFVdp concentrations. This re‐alignment provides a target *ex vivo* tissue‐specific prophylactic concentration that may be translatable to an *in vivo* target TFV concentration to guide dose selection. Topical PrEP product development better informed by these PK/PD models improves the possibility of providing another prophylaxis option for high‐risk populations that prefer an alternative formulation to a daily oral pill.

## Funding

The clinical trial (RMP‐02/MTN‐006) was funded by the pre‐Clinical/Clinical HIV Topical Microbicide Program (U19 AI AI060614) and Microbicide Trials Network Laboratory Center (UM1 AI106707). This secondary analysis was supported in part by the Bill and Melinda Gates Foundation (Contract ID OPP1099837) and the Johns Hopkins University Center for AIDS Research (P30 AI042855). P.J. was supported by grant T32 GM007546 from the National Institute of General Medical Sciences (NIGMS).

## Conflicts of Interest

The authors declared no competing interests for this work.

## Author Contributions

P.J., M.G.C., R.S., K.V., P.A., and C.H. wrote the manuscript. P.J., M.G.C., R.S., K.V., P.A., and C.H. designed the research. P.J., M.G.C., R.S., K.V., N.B., and P.A. performed the research. P.J., M.G.C., R.S., K.V., and N.B. analyzed the data.

## Supporting information

Supplementary Material
