# Supplementary material for: A Mechanistic In Vivo/Ex Vivo Pharmacokinetic‐Pharmacodynamic Model of Tenofovir for HIV Prevention
Source: CPT Pharmacometrics Syst Pharmacol. 2021 Feb 6;10(3):179–87. doi: 10.1002/psp4.12583 (PMC7965838; doi:10.1002/psp4.12583)
Supplement: Supplementary file 1 — Supplementary Material [file PSP4-10-179-s001.pdf]

Figure S1: Visual predictive checks for pharmacokinetic model.

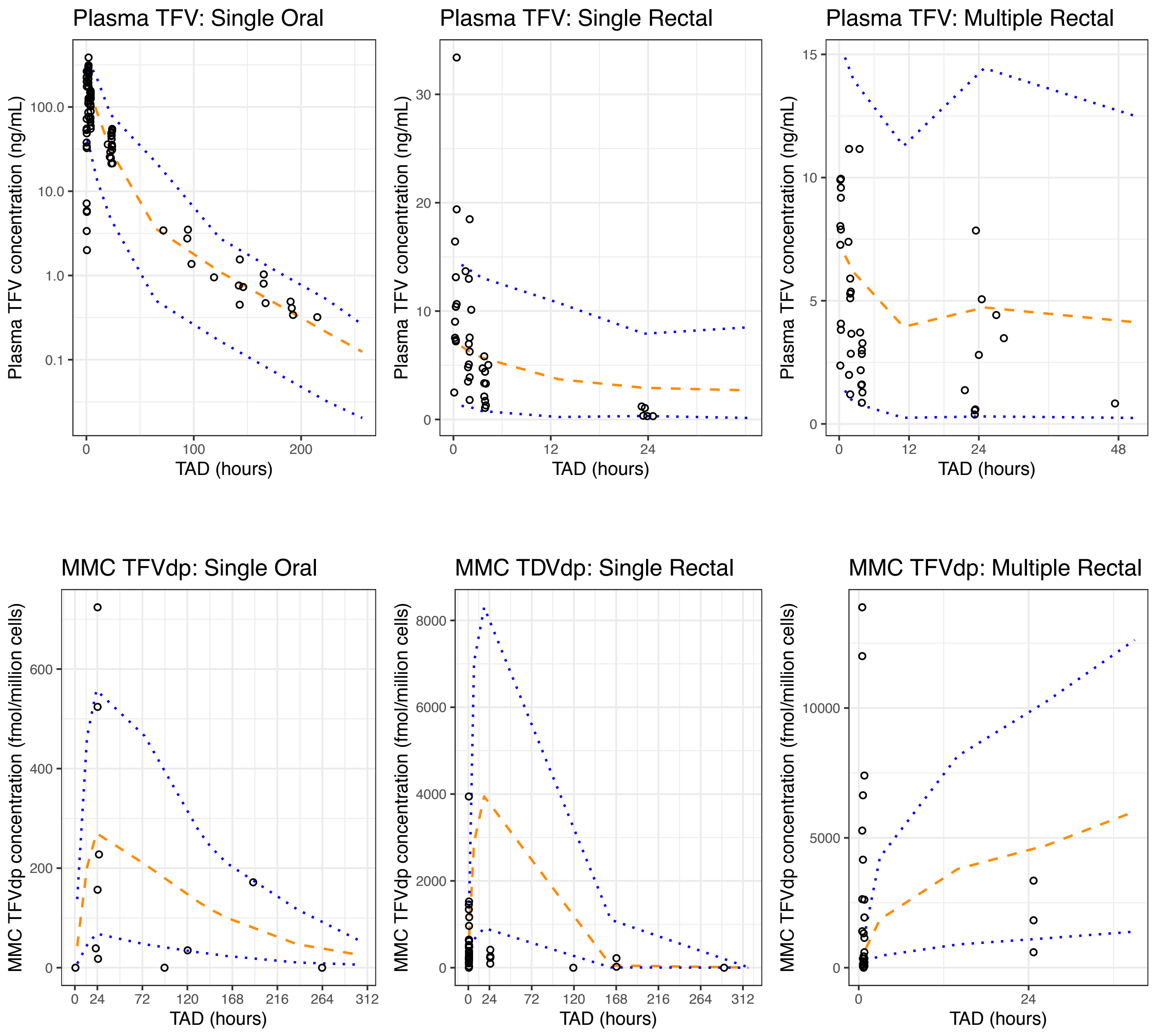

Visual predictive checks comparing parent or metabolite drug concentrations (raw data) and predictions from the pharmacokinetic model stratified by study arm for each matrix. Percentiles of simulated data: upper, dotted, blue line (97.5 for plasma, 90 for MMC); middle, dashed line, orange (50 for plasma and MMC); lower, dotted, blue line (2.5 for plasma, 10 for MMC). Raw data: black open circles. TFV, tenofovir; TFVdp, tenofovir-diphosphate; TAD, time after dose; MMC, rectal mononuclear cells; fmol, femtomole.

***Figure S2: Pharmacodynamic profiles per MMC cell type as a function of TFVdp concentration*.**

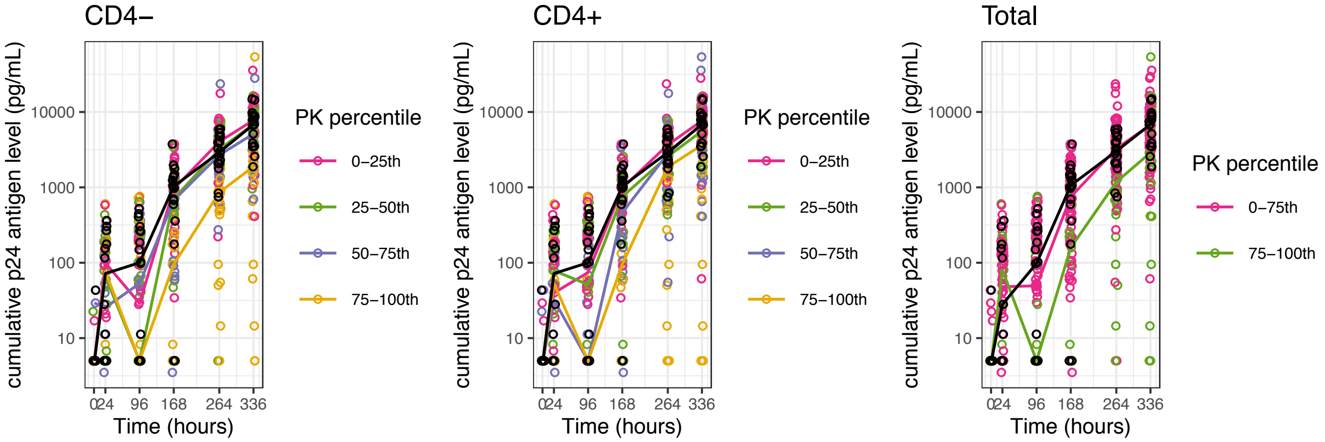

Cumulative p24 antigen expression levels as a function of TFVdp concentration (pharmacokinetic) percentiles stratified by MMC type. Pharmacokinetic percentiles were determined by grouping TFVdp concentrations for each cell type into quantiles (0, 0.25, 0.50, 0.75, 1). Lines: colored, median; black, baseline (no treatment). PK, pharmacokinetic; pg, picogram; mL, milliliter; TFVdp, tenofovir-diphosphate; MMC, rectal mononuclear cell.

***Figure S3: Correlation plot for MMC cell types*.**

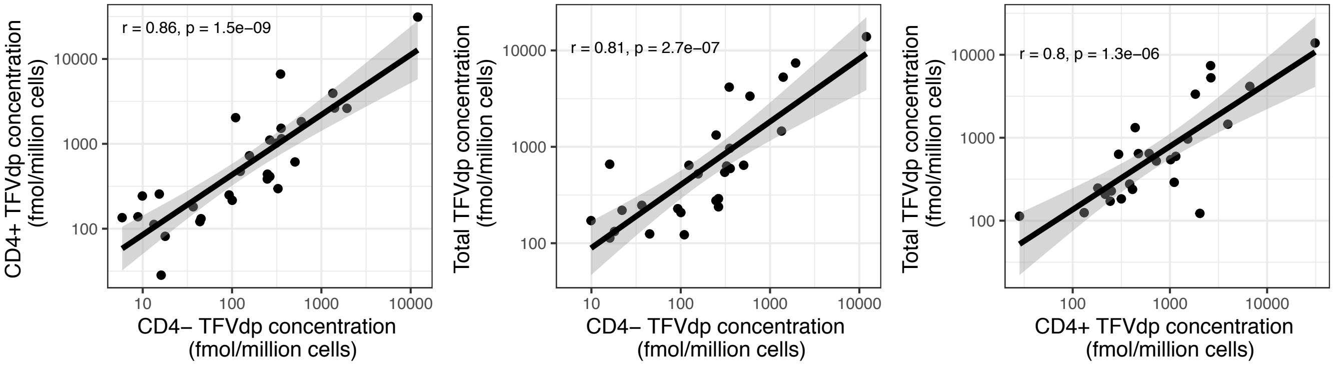

Pearson correlation of metabolite drug concentrations per MMC type. Shadow: grey, 95% confidence interval. TFVdp, tenofovir-diphosphate; fmol, femtomole; MMC, rectal mononuclear cell.

Figure S4: Visual predictive checks for pharmacokinetic-pharmacodynamic model.

Cell type: CD4-

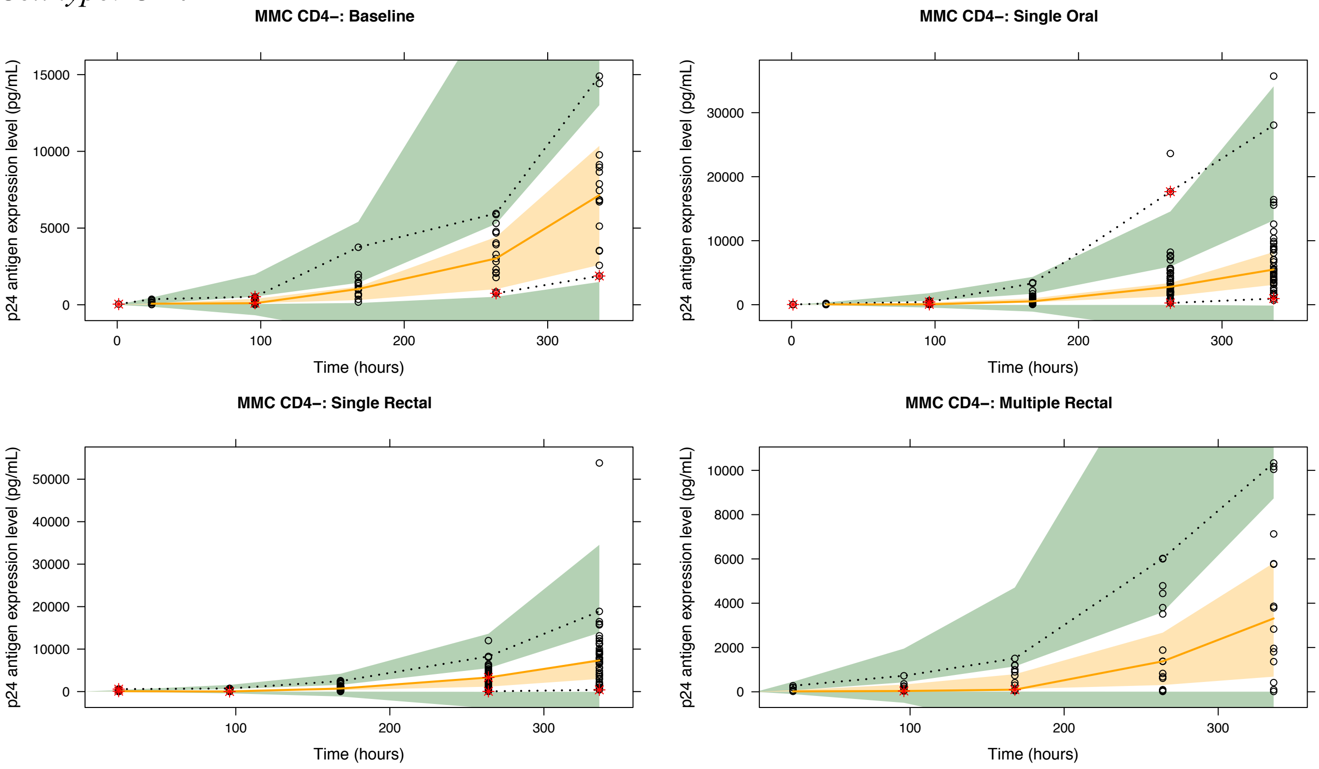

Cell type: CD4+

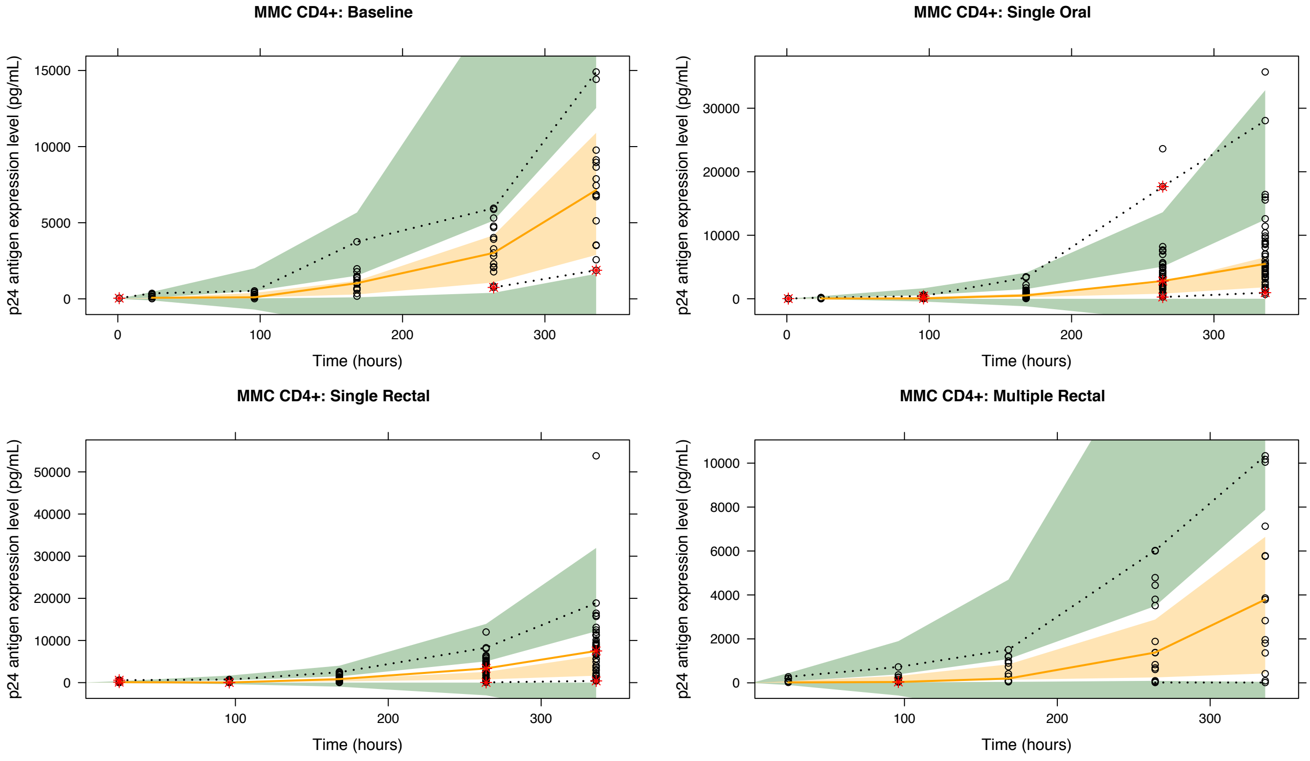

Cell type: total

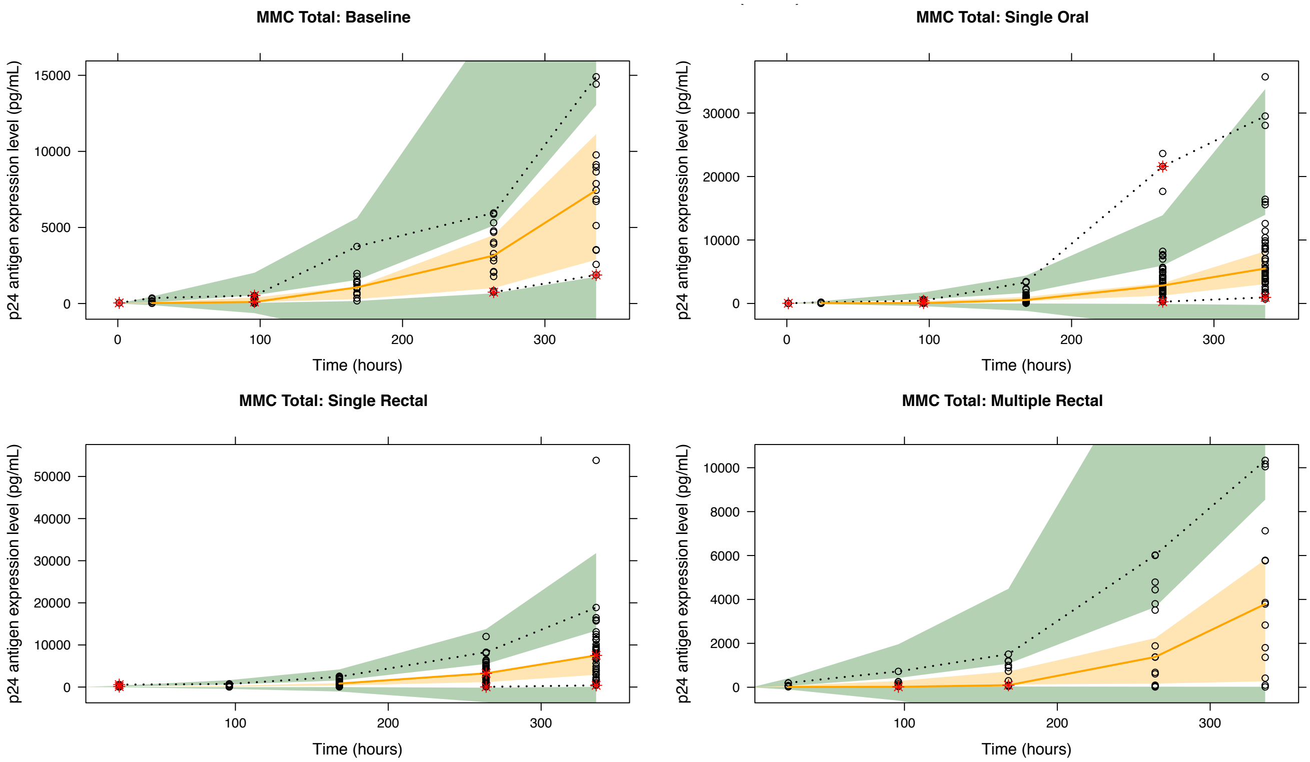

Visual predictive checks comparing parent or metabolite drug concentrations (raw data) and predictions from the pharmacokinetic-pharmacodynamic model stratified by study arm (including baseline) for all 3 cell types ($CD4^-$, CD_4^+, total). Percentiles of raw data: upper, dotted, black (97.5); middle, solid, yellow (50); lower, dotted, black (2.5). Outliers: red stars (*). Raw data: black open circles. Simulation-based confidence intervals: upper, green (97.5); middle, yellow (50); lower, green (2.5).

***Table S1: Description of raw data*.**

Matrix	Parent or Metabolite (cell type)	#Subjects	#Observations (%BLQ)	# Observations/study arm	Concentration Range	Units
Plasma	TFV	18	270 (39)	99 (single oral)<br>97 (single rectal)<br>74 (multiple rectal)	0.310 – 387	ng/mL
Rectal tissue	TFV	18	116 (80)	50 (single oral)<br>49 (single rectal)<br>17 (multiple rectal)	0 – 430	ng/mg
Rectal tissue	TFVdp	18	116 (70)	50 (single oral)<br>49 (single rectal)<br>17 (multiple rectal)	0 – 7,188	fmol/mg
MMC	TFVdp (CD_4^-)	18	104 (75)	45 (single oral)<br>43 (single rectal)<br>16 (multiple rectal)	0 – 12,000	fmol/million cells
MMC	TFVdp (CD_4^+)	15	38 (45)	11 (single oral)<br>16 (single rectal)<br>11 (multiple rectal)	0 – 31,153	fmol/million cells
MMC	TFVdp (Total)	16	47 (47)	14 (single oral)<br>21 (single rectal)<br>12 (multiple rectal)	0 – 13,880	fmol/million cells
PBMC	TFVdp (CD_4^-)	18	50 (94)	39 (single oral)<br>10 (single rectal)<br>1 (multiple rectal)	0 – 62.0	fmol/million cells
PBMC	TFVdp (CD_4^+)	11	16 (100)	8 (single oral)<br>5 (single rectal)<br>3 (multiple rectal)	0 – 0	fmol/million cells
PBMC	TFVdp (Total)	18	113 (91)	59 (single oral)<br>26 (single rectal)<br>28 (multiple rectal)	0 – 38.6	fmol/million cells
--	p24 antigen	18	682 (24)	87 (baseline)<br>260 (single oral)<br>252 (single rectal)<br>83 (multiple rectal)	3.5 – 53,818	pg/mL

Number of subjects and observations (including per study arm), percent of observations deemed below the limit of quantification, and parent or metabolite drug concentration range with units per matrix. p24 antigen level range reflects the cumulative p24 antigen levels obtained across the 14-day *ex vivo* explant assay. PBMC, peripheral blood mononuclear cells; MMC, rectal mononuclear cells; TFV, tenofovir; TFVdp, tenofovir-diphosphate; BLQ, below assay lower limit of quantification; fmol, femtomole; ng, nanogram; mg, milligram; pg, picogram; mL, milliliter.

Table S2: Pharmacokinetic-pharmacodynamic baseline model.

Parameter	Population Estimate (RSE, %)	Inter-individual Variability, %CV (RSE, %)
k_{growth} [/h]	0.0320*	-
k_{death} [/h]	0.0193 (5)	-
k_{p24} [/h]	0.00400*	72.5 (24)
Ratio [pg/virions]	0.0404 (27)	-
Proportional error [%CV]	40.4 (17)	
Additive error [pg/mL]	111 (28)	
OFV	1180	

**Estimate fixed to value from literature*

Parameter estimates for pharmacokinetic-pharmacodynamic baseline (no treatment) model with objective function value. k_g, growth rate; k_d, death rate; k_p, p24 antigen expression rate; RSE, relative standard error; %CV, percent coefficient of variation; pg, picogram; mL, milliliter; fmol, femtomole; OFV, objective function value.

***Table S3: Pharmacokinetic-pharmacodynamic model summary*.**

Model Description	Objective Function Value (dOFV)		
	CD_4^-	CD_4^+	TOTAL
Treatment effect	8280	8164	8140
PKPD effect	8168 (-112)	8105 (-59)	8040 (-100)
Drug degradation effect	8161 (-7)	8097 (-8)	8032 (-8)

Objective function values for each model iteration (treatment effect, PKPD effect, drug degradation effect). dOFV, difference in objective function value relative to previous model.

Supplementary Model Code 1: Pharmacokinetic model code. Annotated NONMEM code for multicompartment PK model; limited to key matrices (plasma, MMC).

```
;;Model Description: MMC TFVdp and Plasma TFV PK Model

$PROBLEM run1_PK.mod

$INPUT    ID TIME TAD ROUTE AMT ADDL II PARENTORMET MDV EVID MATRIX VISIT ARM CELL DV BLQ CMT DSET

$DATA data_PK.csv
IGNORE=@
IGNORE=(TAD.LT.0)                                    ; ignore time after dose less than zero
IGNORE=(BLQ.EQ.1)                                    ; ignore concentrations below the lower limit of quantification
IGNORE=(CMT.GT.4)                                    ; ignore tissue and PBMC compartments

$SUBROUTINES ADVAN13 TOL=9

$MODEL
NCOMP=4                                              ; 2 compartment plasma model with oral absorption and effect compartment for MMC
COMP=(DEPOT, DEFDOSE)                                ; absorption compartment
COMP=(CENTRAL, DEFOBS)                               ; plasma central compartment
COMP=(PERIPH)                                        ; plasma peripheral compartment
COMP=(MMC)                                           ; MMC compartment

;---------------- UNITS -------------------------

;TIME      [hr]
;DV        [ng/mL; plasma] [fmol/million cells; MMC]
;AMT       [mg]

$PK

;---------------- PLASMA PK PARAMETERS-------------------------

KA = THETA(3)*EXP(ETA(1))                            ; oral absorption rate constant
IF (ROUTE.EQ.2) KA = THETA(8)*EXP(ETA(5))            ; rectal absorption rate constant
CL = THETA(4)*EXP(ETA(2))                            ; clearance
V2 = THETA(5)*EXP(ETA(3))                            ; central volume
Q  = THETA(6)*EXP(ETA(4))                            ; intercompartmental clearance
V3 = THETA(7)*EXP(ETA(6))                            ; peripheral volume
F1 = 1                                               ; oral bioavailability
IF (ROUTE.EQ.2) F1 = THETA(9)                        ; rectal bioavailability

MXSTEP=50000

S2 = V2/1000
K20 = CL/V2                                          ; elimination rate constant from central compartment
K23 = Q/V2                                           ; rate constant from central to peripheral compartment
K32 = Q/V3                                           ; rate constant from peripheral to central compartment

;---------------- MMC PK PARAMETERS----------------

KPMMC = THETA(12)*EXP(ETA(7))                        ; Ko_PMMC
                                                     ; oral rate constant for drug moving between plasma and MMC compartment

IF (ROUTE.EQ.2) KPMMC = THETA(13)*EXP(ETA(8))        ; Kr_PMMC
                                                     ; rectal rate constant for drug moving between plasma and MMC compartment

RPMMC = THETA(14)*EXP(ETA(9))                        ; Ro_PMMC
                                                     ; oral ratio of drug concentration in MMC/plasma compartment

IF (ROUTE.EQ.2) RPMMC = THETA(15)*EXP(ETA(10))       ; Rr_PMMC
                                                     ; rectal ratio of drug concentration in MMC/plasma compartment


;---------------- ODES --------------------------------------

$DES
DADT(1) = -KA*A(1)                                   ; ODE for absorption compartment
DADT(2) =  KA*A(1) - K23*A(2) + K32*A(3) - K20*A(2)  ; ODE for plasma central compartment
DADT(3) =  K23*A(2) - K32*A(3)                       ; ODE for plasma peripheral compartment
```

```
CP=0                                        ; initial condition
IF(A(2).GT.0) CP = A(2)/S2                  ; drug concentration in plasma central compartment
DADT(4) = KPMMC*(RPMMC*CP-A(4))             ; ODE for MMC compartment

;----------------- INITIAL ESTIMATES --------------------------

$THETA
(0.515)     FIX     ; 1 proportional error - plasma oral
(0)         FIX     ; 2 additive error - plasma oral
(0, 1.78)   FIX     ; 3 KA_oral [1/hr]
(0, 52.1)   FIX     ; 4 CL [L/hr]
(0, 679)    FIX     ; 5 V2 [L]
(0, 7.9)    FIX     ; 6 Q [L/hr}
(0, 399)    FIX     ; 7 V3 [L]
(0, 22.2)   FIX     ; 8 KA_rectal [1/hr]
(0, 0.102)  FIX     ; 9 F1_rectal
(0, 0.52)   FIX     ; 10 proportional error - plasma rectal
(0, 3.71)   FIX     ; 11 additive error - plasma rectal
(0, 0.0102)         ; 12 Ko_PMMC [1/hr]
(0, 0.1)            ; 13 Kr_PMMC [1/hr]
(0, 11.4)           ; 14 Ro_PMMC [(fmol/million cells)/(ng/mL)]
(0, 1530)           ; 15 Rr_PMMC [(fmol/million cells)/(ng/mL)]
(0, 1.33)           ; 16 proportional error - MMC
(0)         FIX     ; 17 additive error - MMC

$OMEGA
 0.262      FIX     ; 1 ETA KA_oral
 0          FIX     ; 2 ETA CL
 0.045      FIX     ; 3 ETA V2
 0          FIX     ; 4 ETA Q
 0          FIX     ; 5 ETA KA_rectal
 0          FIX     ; 6 ETA V3
 0          FIX     ; 7 ETA Ko_PMMC
 0          FIX     ; 8 ETA Kr_PMMC
 0          FIX     ; 9 ETA Ro_PMMC
 0          FIX     ; 10 ETA Rr_PMMC

$SIGMA
 1          FIX

;----------------- PREDICTION & ERROR MODEL --------------------------

$ERROR
IPRED = A(2)/S2                                                    ; plasma compartment [ng/mL]
IF(CMT.EQ.4) IPRED = A(4)                                          ; MMC compartment [fmol/million cells]
IRES = DV-IPRED

W = SQRT(THETA(2)**2+(THETA(1)*IPRED)**2)                          ; combined error model (additive + proportional) - plasma oral
IF (ROUTE.EQ.2)     W = SQRT(THETA(11)**2+(THETA(10)*IPRED)**2)    ; combined error model (additive + proportional) - plasma rectal
IF (CMT.EQ.4)       W = SQRT(THETA(17)**2+(THETA(16)*IPRED)**2)    ; combined error model (additive + proportional) - MMC
IF (W.EQ.0) W = 1

IWRES = IRES/W
Y = IPRED+W*ERR(1)

$EST METHOD=1 INTERACTION MAXEVAL=9999 PRINT=5 NOABORT POSTHOC NSIG=3 SIGL=9
$COV PRINT=E UNCONDITIONAL

$TABLE ID TIME TAD ROUTE AMT ADDL II PARENTORMET MDV EVID MATRIX VISIT ARM CELL DV BLQ CMT DSET CL KA V2 V3 Q
K20 K23 K32 RPMMC KPMMC ETA1 ETA2 ETA3 ETA4 ETA5 ETA6 ETA7 ETA8 ETA9 ETA10 IPRED IWRES CWRES NOPRINT ONEHEADER
FILE=sdtab1
```

Supplementary Model Code 2: Pharmacokinetic-pharmacodynamic model code. Annotated NONMEM code for mechanistic PKPD model; limited to a single cell type (total).

```
;; Model Description: PKPD Mechanistic Model

$PROBLEM run1_PKPD.mod

$INPUT    ID VISIT CELL CP FLAG_PK TIME A B C D AVGP24 DV ARM EVID BLQ DOSE

$DATA data_PKPD.csv
IGNORE=@
IGNORE=(CELL.EQ.4)                          ; ignore CD4- cells
IGNORE=(CELL.EQ.5)                          ; ignore CD4+ cells

$SUBROUTINES ADVAN13 TOL=12

$MODEL
NCOMP=2
COMP=(HIV)                                  ; HIV compartment
COMP=(P24)                                  ; p24 compartment

;---------------- UNITS -------------------------

;TIME      [hr]
;DV        [pg/mL]
;Longitudinal PK concentration (MMC) included in dataset (CP column) [fmol/million cells]

$PK

;---------------- DISEASE PARAMETERS-------------------------

KG = THETA(3)*EXP(ETA(1))                   ; rate constant for virus growth
KD = THETA(4)*EXP(ETA(2))                   ; rate constant for virus death
KP = THETA(5)*EXP(ETA(3))                   ; rate constant for p24 antigen expression
A_0(1) = THETA(6)*EXP(ETA(4))               ; initial condition, HIV compartment
A_0(2) = 0                                  ; initial condition, p24 compartment
RATIO = THETA(7)*EXP(ETA(5))                ; ratio of HIV viral load to p24 antigen level

;---------------- PKPD PARAMETERS-----------------------------

SL = THETA(8)*EXP(ETA(6))                   ; slope of linear effect model
KDEG = THETA(9)*EXP(ETA(7))                 ; rate constant for drug degradation

;---------------- ODES -------------------------------------

$DES
CPP = CP*EXP(-KDEG*T)                       ; PK drug degradation expression
E = 0
IF(VISIT.NE.2) E = SL*CPP                   ; linear drug effect expression after baseline visit

DADT(1) = KG*A(1)*(1-E) - KD*A(1)           ; ODE for HIV compartment
DADT(2) = KP*(RATIO*A(1) - A(2))            ; ODE for p24 compartment

;----------------- INITIAL ESTIMATES --------------------------

$THETA
(0, 0.880)                                  ; 1 proportional error
(0, 3.62)                                   ; 2 additive error
(0.032)     FIX                             ; 3 KG [1/hr]
(0, 0.0193) FIX                             ; 4 KD [1/hr]
(0, 0.004)  FIX                             ; 5 KP [1/hr]
(10000)     FIX                             ; 6 initial condition for A(1)
(0, 0.0404) FIX                             ; 7 RATIO [pg/virions]
(0, 0.000085)                               ; 8 SL [(pg/mL)/(fmol/million cells)]
(0, 0.0018) FIX                             ; 9 KDEG [1/hr]

$OMEGA
 0          FIX                             ; 1 ETA KG
 0          FIX                             ; 2 ETA KD
 0.525      FIX                             ; 3 ETA KP
 0          FIX                             ; 4 ETA A_0(1)
```

```
 0        FIX                         ; 5 ETA RATIO
 0        FIX                         ; 6 ETA SL
 0        FIX                         ; 7 ETA KDEG

$SIGMA
 1        FIX

;----------------- PREDICTION & ERROR MODEL --------------------------

$ERROR
IPRED = A(2)                          ; p24 compartment [pg/mL]
IRES = DV-IPRED

W = SQRT(THETA(2)**2+(THETA(1)*IPRED)**2)   ; combined error model (additive + proportional)
IF (W.EQ.0) W = 1

IWRES = IRES/W
Y = IPRED+W*ERR(1)

$EST METHOD=1 INTERACTION MAXEVAL=9999 PRINT=5 NOABORT POSTHOC NSIG=3 SIGL=9
$COV PRINT=E UNCONDITIONAL

$TABLE ID VISIT CELL CP FLAG_PK TIME A B C D AVGP24 DV ARM EVID BLQ DOSE KG KD KP RATIO SL KDEG ETA1 ETA2 ETA3 ETA4
ETA5 ETA6 ETA7 IPRED IWRES CWRES NOPRINT ONEHEADER FILE=sdtab1
```
